# Supplementary material for: Resveratrol attenuates non-steroidal anti-inflammatory drug-induced intestinal injury in rats in a high-altitude hypoxic environment by modulating the TLR4/NFκB/IκB pathway and gut microbiota composition
Source: PLoS One. 2024 Aug 12;19(8):e0305233. doi: 10.1371/journal.pone.0305233 (PMC11318858; doi:10.1371/journal.pone.0305233)
Supplement: S1 Table — (DOCX) [file pone.0305233.s001.docx]

**S1 Table** Reuter's scale

The two main evaluation criteria are ulceration and adhesion.

Ulcer scale: **0** is no ulcer;

**1** is localized congestion of the small bowel, however, no ulcer is seen;

**2** is ulcer of the small bowel without congestion or thickening of the bowel wall;

**3** is ulceration with inflammation in one site;

**4** is ulceration or inflammation in two or more sites;

**5** is the presence of a perforation of the small bowel.

Adhesions: **0** is no adhesions;

**1** is mild adhesions;

**2** is severe adhesions.

The sum of the ulcer score and adhesion score was the total score.
