# Supplementary material for: Resveratrol attenuates non-steroidal anti-inflammatory drug-induced intestinal injury in rats in a high-altitude hypoxic environment by modulating the TLR4/NFκB/IκB pathway and gut microbiota composition
Source: PLoS One. 2024 Aug 12;19(8):e0305233. doi: 10.1371/journal.pone.0305233 (PMC11318858; doi:10.1371/journal.pone.0305233)
Supplement: S2 Table — (DOCX) [file pone.0305233.s002.docx]

**S2 Table** Chiu 's scale

| Score | Mucosal condition of the small intestine |
| --- | --- |
| 0 points | Normal intestinal mucosal villi |
| 1 points | Mild subepithelial edema, capillary dilatation and congestion of the apical intestinal mucosa |
| 2 points | Intestinal mucosa with enlarged subepithelial spaces, moderate edema of the lamina propria, and dilated central celiac ducts |
| 3 points | Cellular degeneration and necrosis of the epithelial layer of the intestinal mucosa, marked edema of the lamina propria, and apical detachment of a few villi |
| 4 points | Degeneration, necrosis, detachment of the epithelial cell layer of the intestinal mucosa, exposure of the lamina propria, dilatation and congestion of the capillaries, and detachment of some villi |
| 5 points | Shedding of intestinal mucosal villi, disintegration of lamina propria, bleeding or ulcer formation |
