# Supplementary figures and images for: Resveratrol attenuates non-steroidal anti-inflammatory drug-induced intestinal injury in rats in a high-altitude hypoxic environment by modulating the TLR4/NFκB/IκB pathway and gut microbiota composition
Source: PLoS One. 2024 Aug 12;19(8):e0305233. doi: 10.1371/journal.pone.0305233 (PMC11318858; doi:10.1371/journal.pone.0305233)

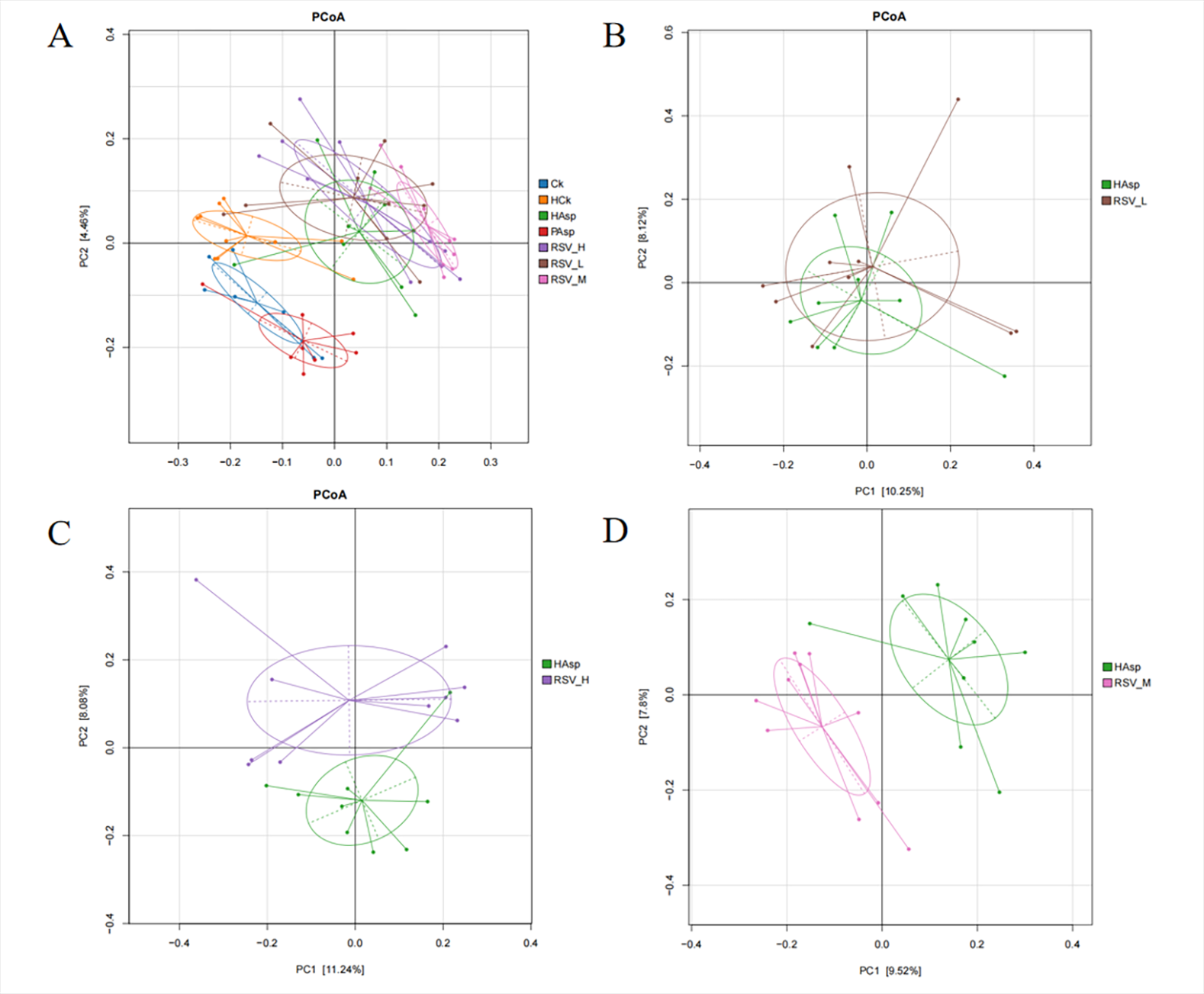

Supplement: S1 Fig — (A) PCoA analysis of different treatment groups; (B) HAsp vs RSVL; (C) HAsp vs RSVM; (D)HAsp vs RSVH. (TIF) [file pone.0305233.s003.tif]

Occludin

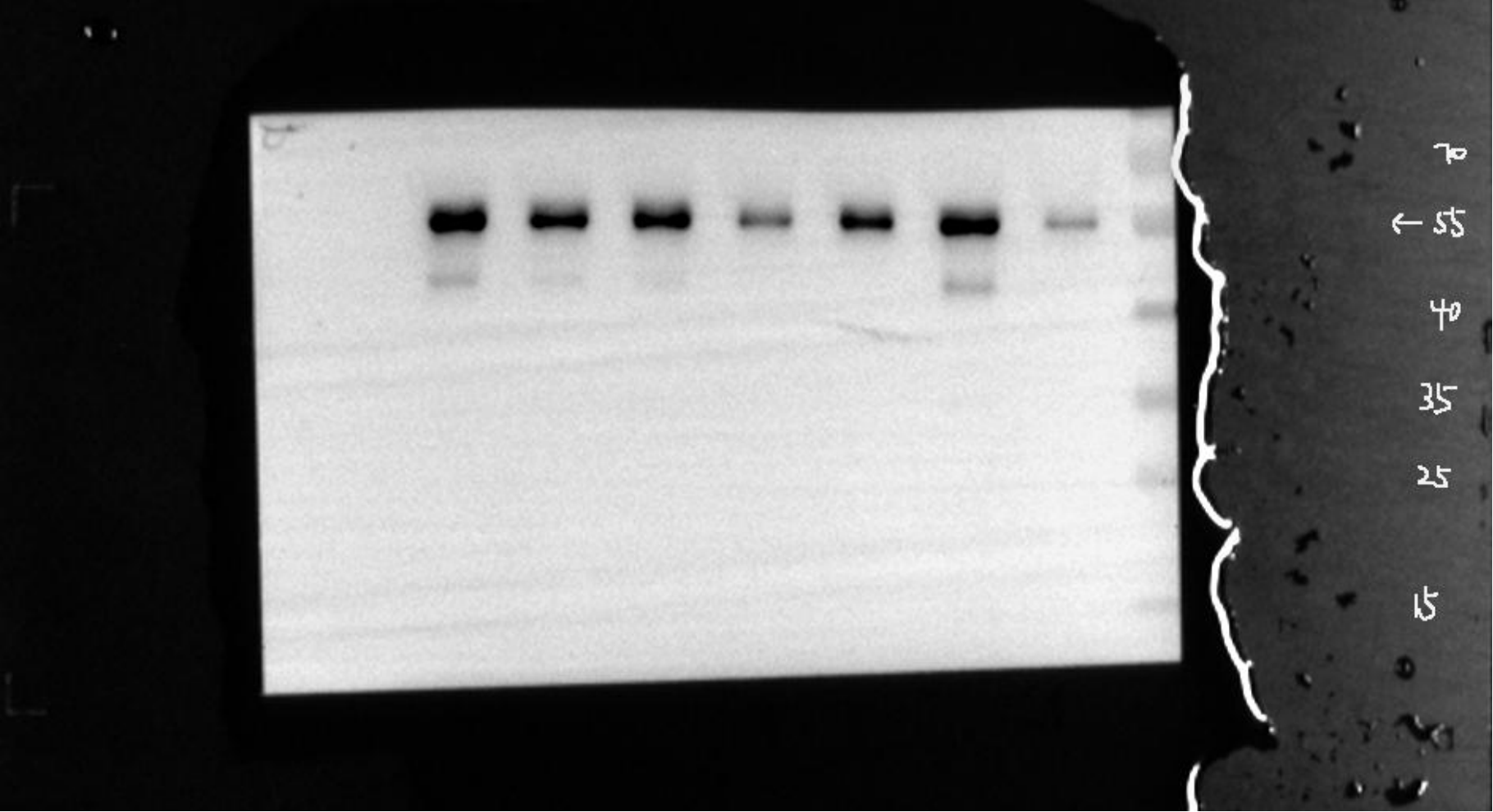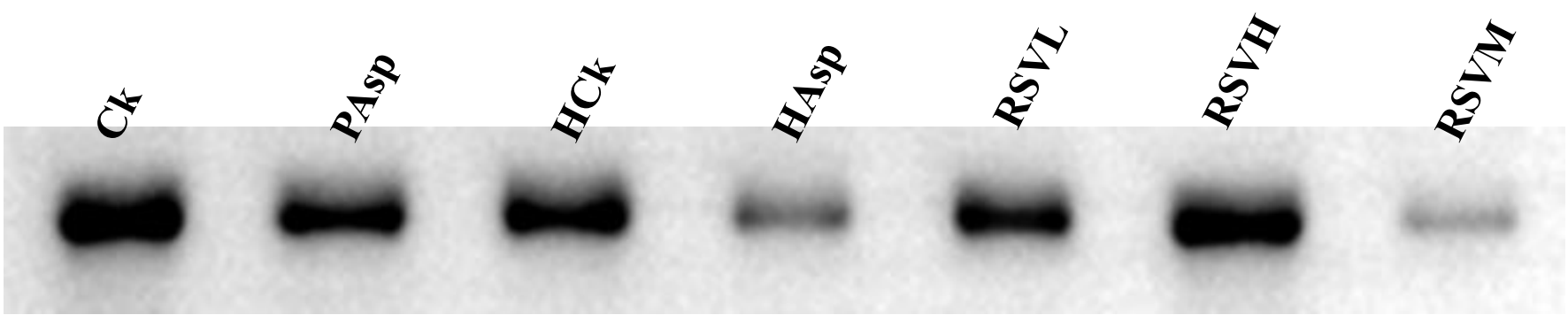

ZO-1

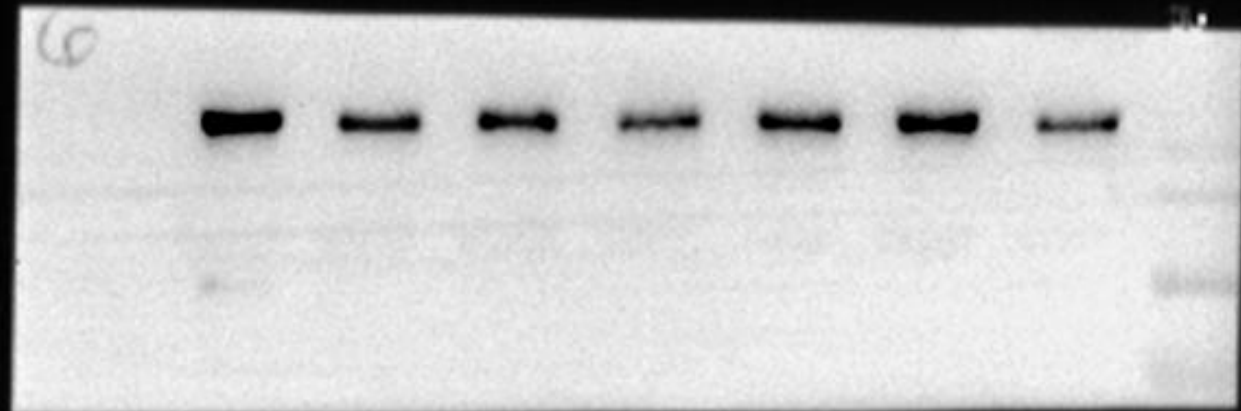

180  
130  
100

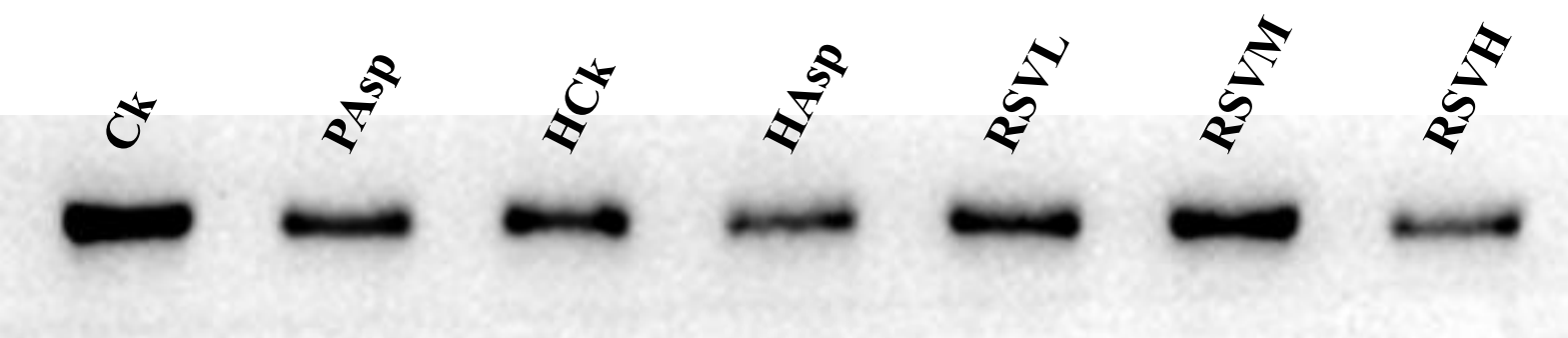

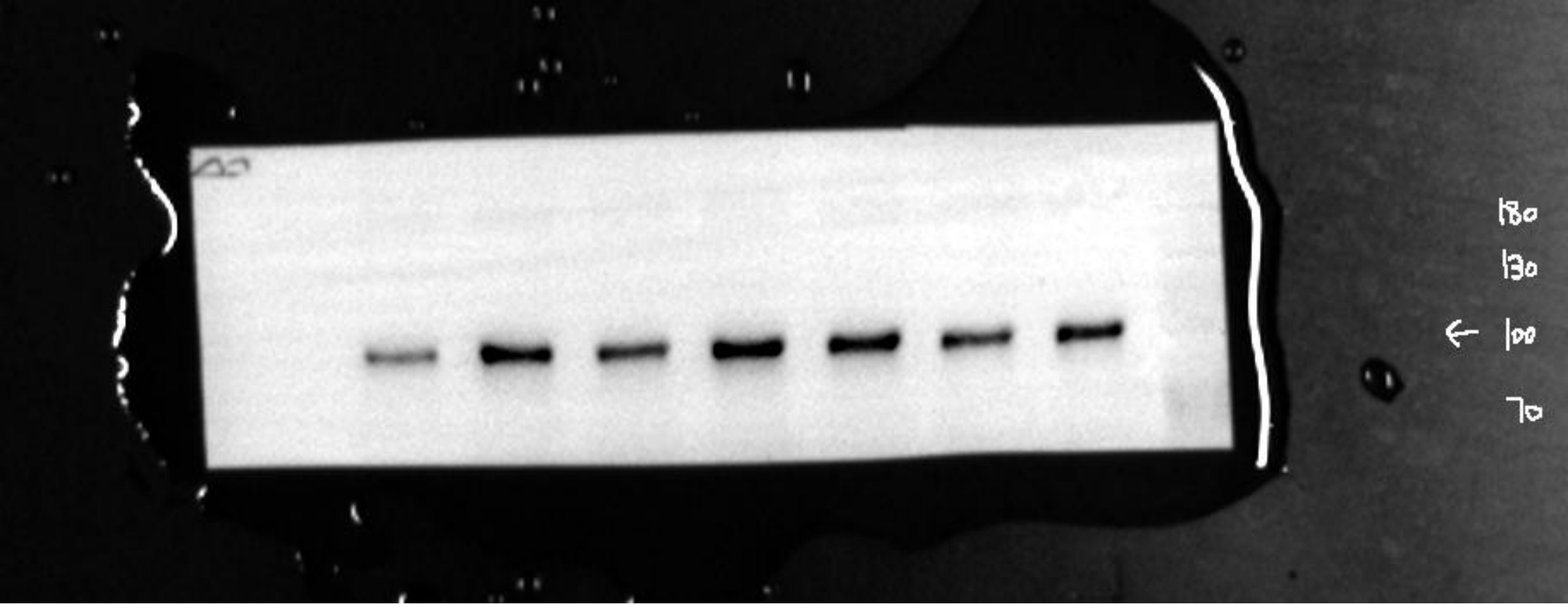

**TLR4**

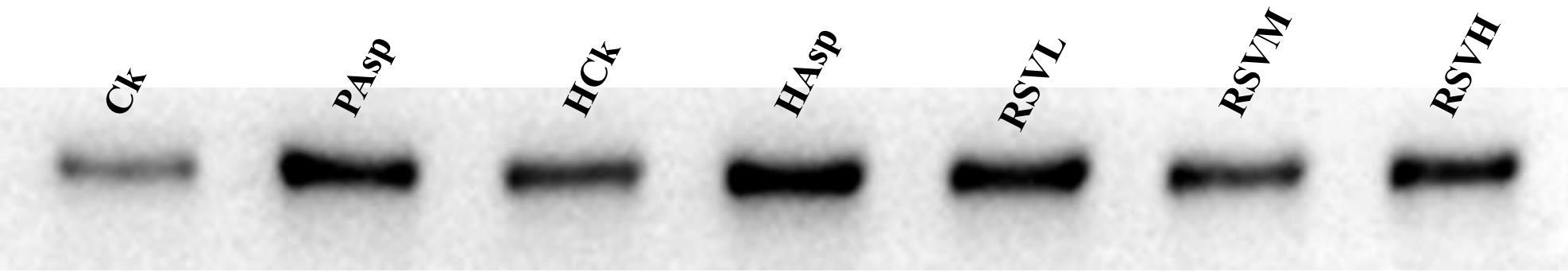

I $\kappa$ B

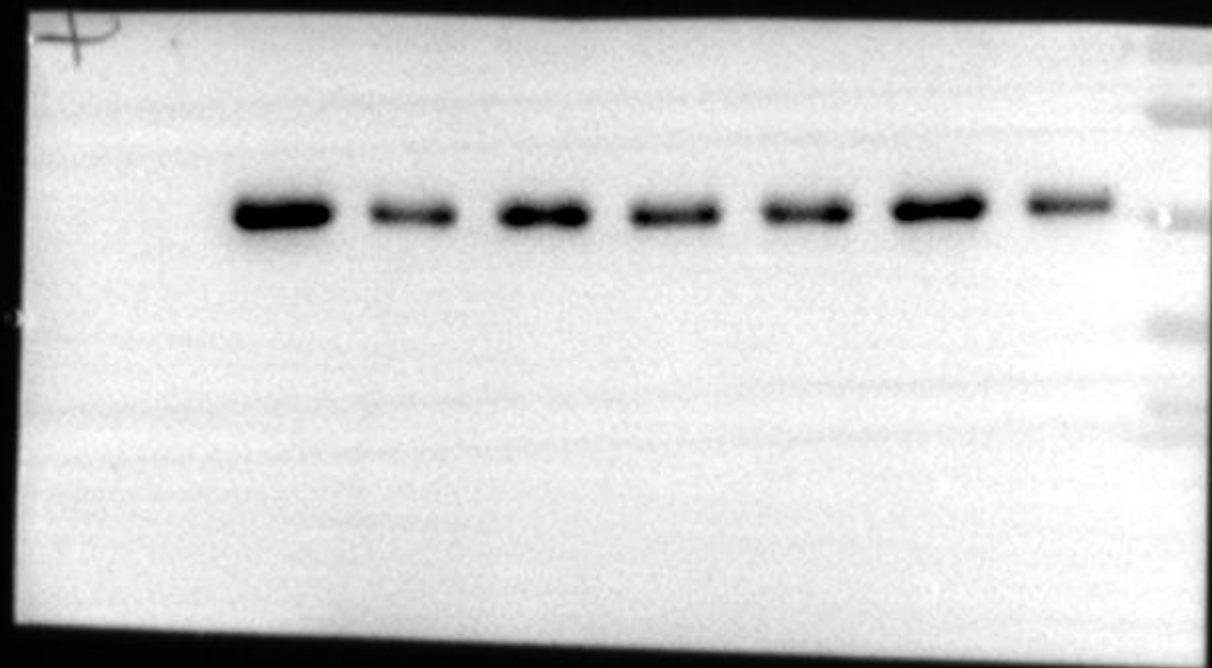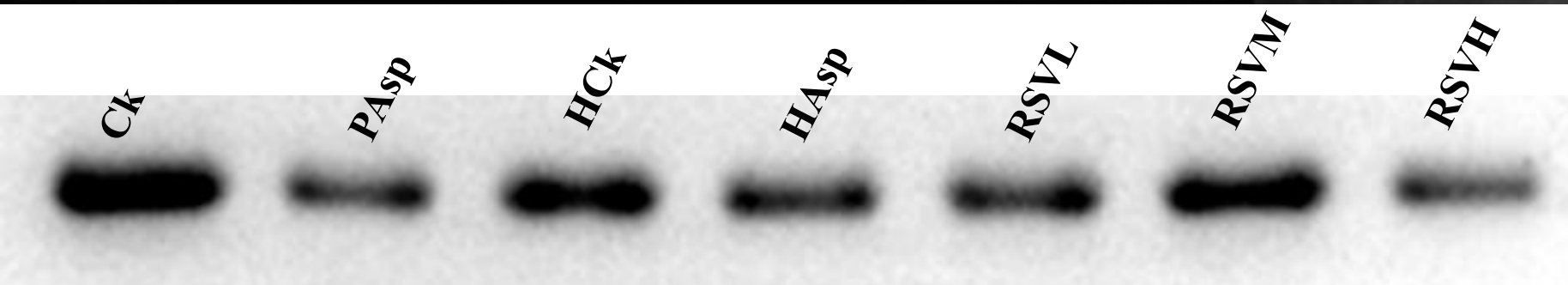

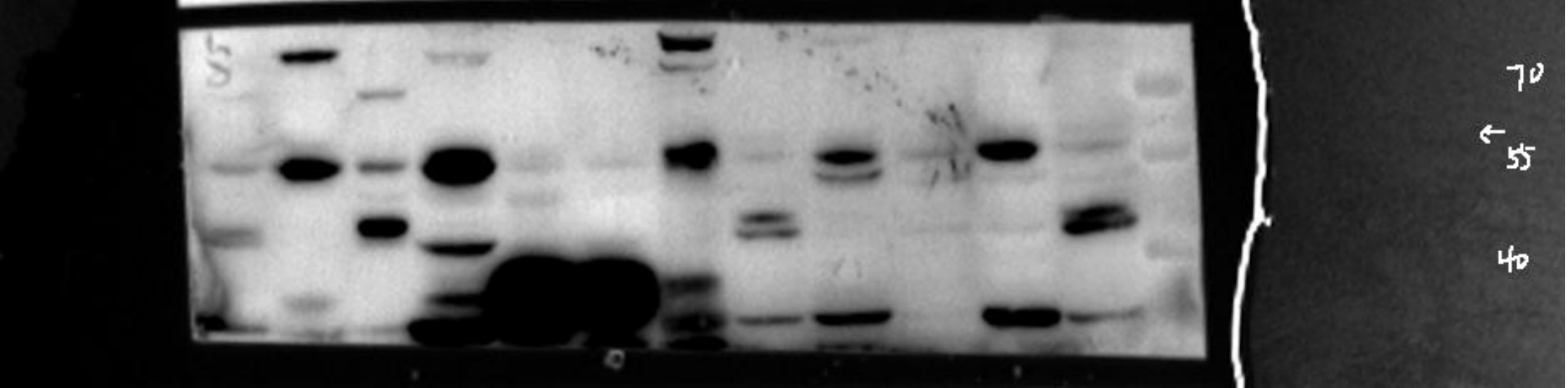

NF-κB

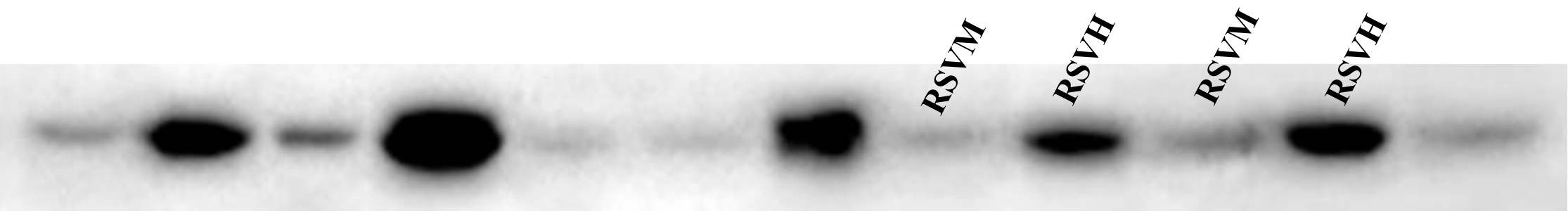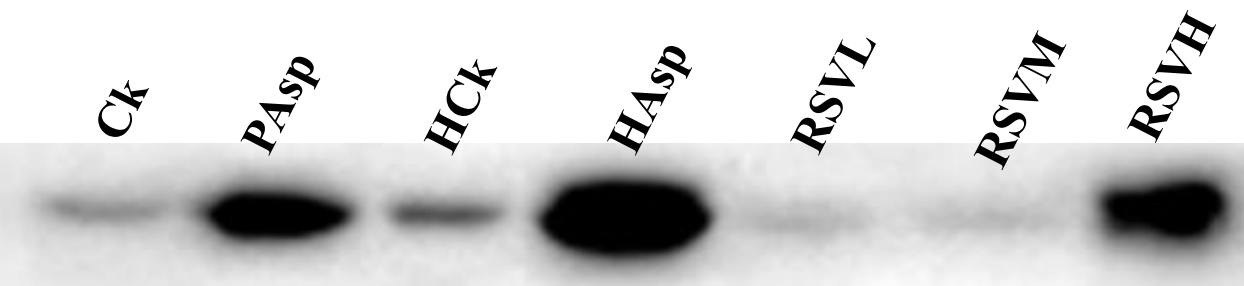

$\beta$ -actin

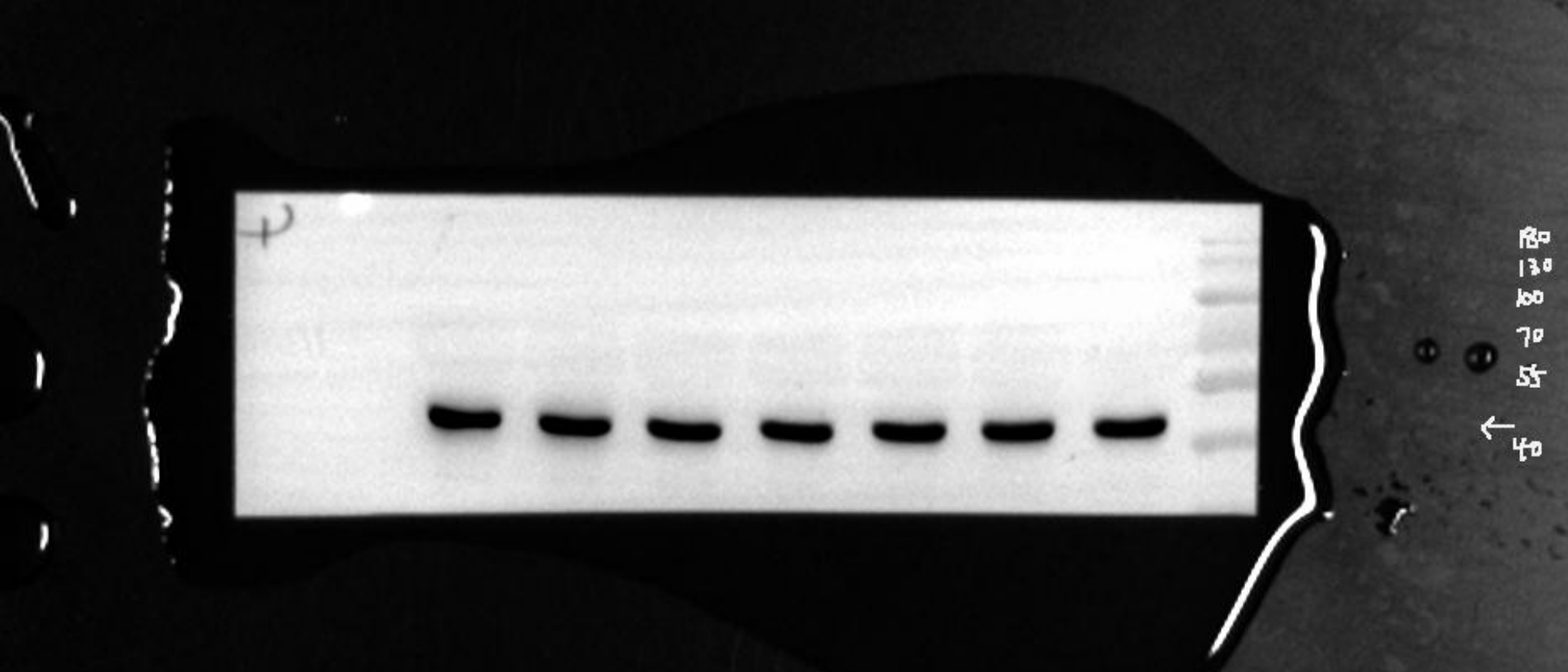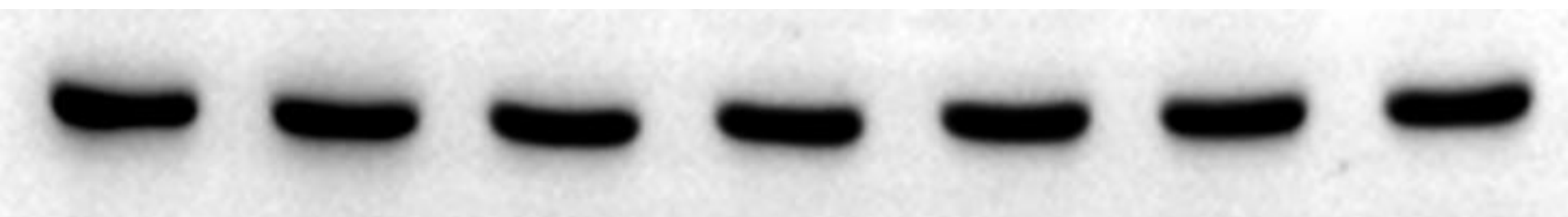

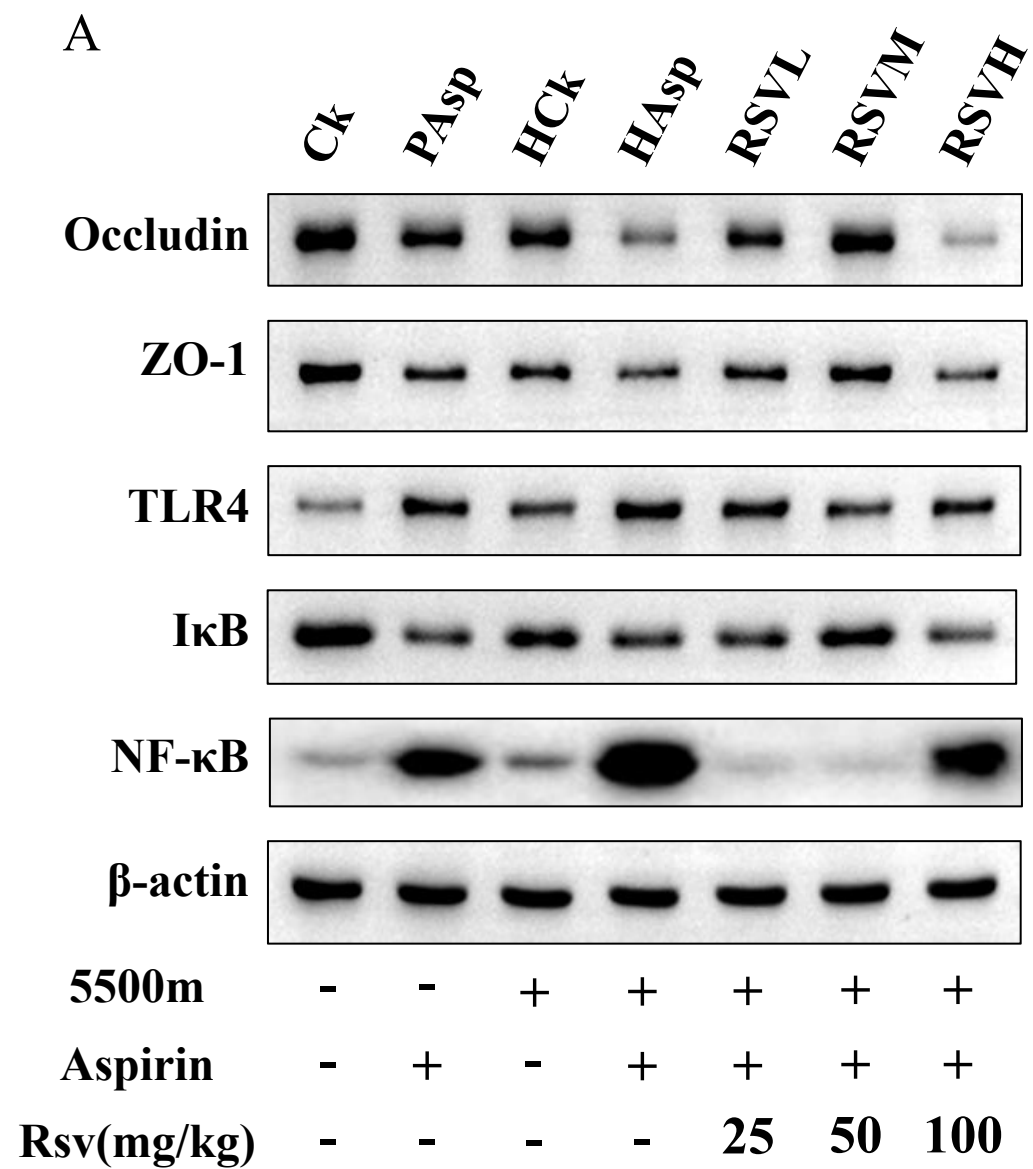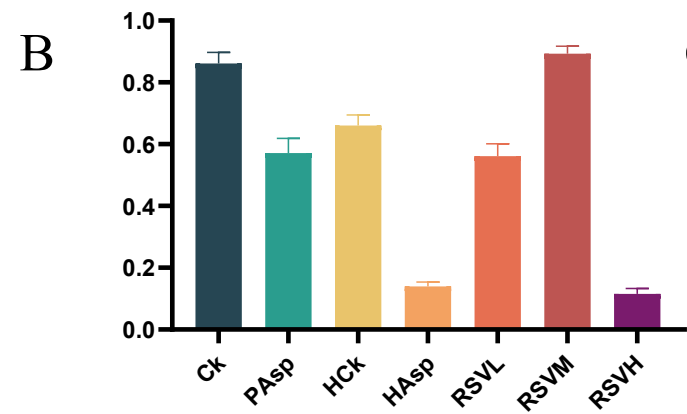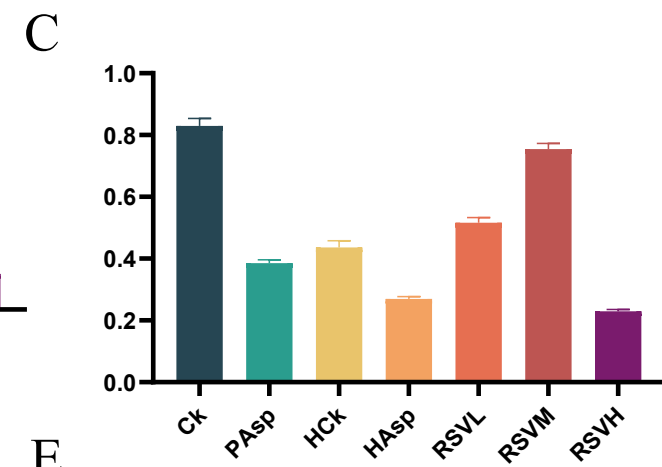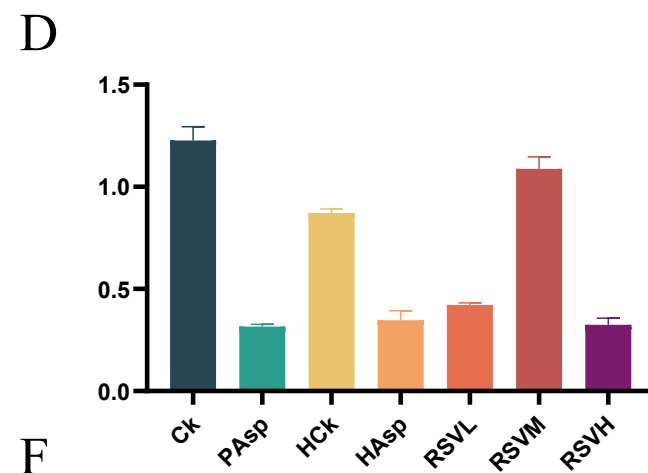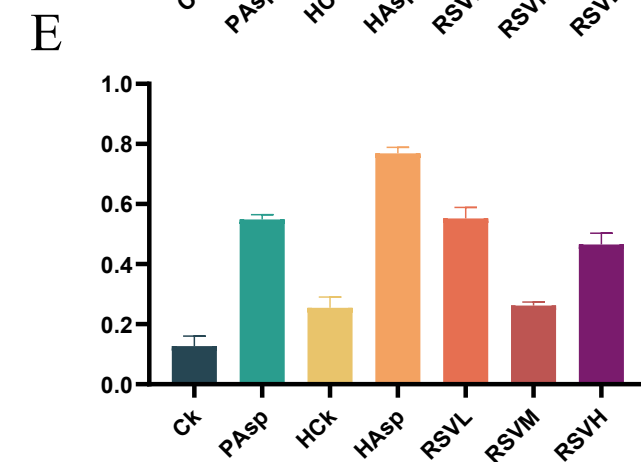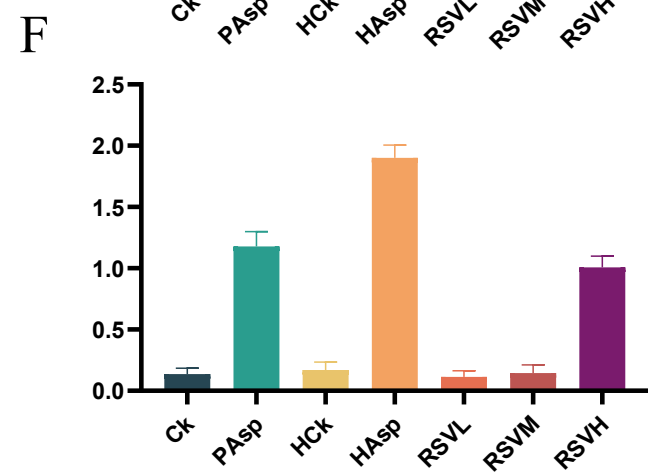

Supplement: S1 Raw images — (PDF) [file pone.0305233.s004.pdf]
